# Operational margin of high-school tennis tournaments under heat constraints: a wet-bulb globe temperature-based scenario analysis

**DOI:** 10.3389/fspor.2026.1914945

**Published:** 2026-10-09

**Authors:** Hiroki Yamaguchi, Takaaki Mori, Hiromi Hanano, Kan Oishi, Kentaro Ikeue, Chie Omichi, Kojiro Ishii

**Affiliations:** 1 Graduate School of Health and Sports Science, Doshisha University, Kyotanabe, Kyoto, Japan; 2 Research Fellow, Japan Society for the Promotion of Sciences, Chiyoda-ku, Tokyo, Japan; 3 Faculty of Health and Sports Science, Doshisha University, Kyotanabe, Kyoto, Japan; 4 Faculty of Education, Saga University, Saga, Japan; 5 Center for Learning Support and Faculty Development, Doshisha University, Kyoto, Japan

**Keywords:** environment, heat safety, heat stress mitigation, heat stroke, tennis tournament

## Abstract

**Introduction:**

Environmental heat indices such as wet-bulb globe temperature (WBGT) does not directly indicate whether a tournament schedule can be completed. Therefore, in this study, we aimed to translate WBGT-based environmental conditions into operational indicators relevant to tournament management and evaluate the effects of tournament scheduling and timing on tennis tournament feasibility.

**Methods:**

In this observational study, WBGT data (April–October 2018–2022; *n* = 119,048) from 11 Japan Meteorological Agency stations were analyzed. Hours with WBGT <28 were defined as low-risk operational time and converted into tournament indicators, including feasible tournament days and match capacity. For July–September, the effects of dates (1–10, 11–20, 21–end) and timing (standard, nighttime, and early morning models) were assessed using logistic regression. Scenario analyses combining these conditions were conducted, and the feasibility of the school tennis tournament was evaluated based on low-risk operational time and match capacity.

**Results:**

Adjusting timing significantly increased low-risk operational time (nighttime model: odds ratio, 1.46; 95% confidence interval: 1.42–1.50; early morning model: odds ratio, 1.36; 95% confidence interval: 1.33–1.40). By contrast, shifting the tournament dates alone showed smaller effects. The combined adjustments increased the number of feasible days by up to 0.8 d. Although the predefined operational feasibility threshold was met on average in most scenarios, the magnitude of the operational margin varied among scenarios.

**Conclusion:**

Tournament feasibility should be evaluated based on operational margins as well as the predefined operational feasibility threshold. Although the threshold was met on average in most scenarios, the magnitude of the operational margin varied among tournament conditions. Combining changes in tournament dates with timing adjustments resulted in the greatest increase in low-risk operational time and tournament feasibility among the scenarios examined.

## 1 Introduction

Global average temperatures continue to rise due to climate change, with Japan reporting a temperature increase of 1.44 °C per century (1). The progression of climate change has significantly affected sports environments. Exercise in hot environments not only impairs performance but also increases the risk of heat-related illnesses. Exertional heat stroke is the second leading cause of non-traumatic death among competitive athletes (2), necessitating appropriate assessment and countermeasures against hot environments. The wet-bulb globe temperature (WBGT), which is calculated from the wet-bulb, black globe, and dry-bulb temperatures, is widely used to assess environmental heat stress in sports. It has been adopted in the International Olympic Committee consensus statement (3) and the heat policies of various international sports federations (4, 5) and is commonly used to guide decisions on tournament continuation.

Previous studies on the WBGT in sports environments have progressed in three ways. The first is an assessment of heat stress risks during the tournament. For example, a study on tennis reported that as WBGT increased, the number of requests for cooling devices, calls for medical staff and trainers, and withdrawals due to heatstroke also increased (6). The second is the examination of environmental conditions for specific events. A report by the Ministry of the Environment on venues for the Tokyo Olympic and Paralympic Games presented daily and hourly WBGT distributions, indicating that all venues faced high heat stress risks, particularly between 09:00 and 15:00 during the Olympic period (7). Furthermore, a predictive study on marathons indicated that even with the initially planned 08:30 start time, high heat stress risks were anticipated, suggesting the need to move the start time to early morning (8). Additionally, a study on host cities for the 2026 FIFA World Cup found that at many venues, periods with WBGT exceeding 28 were observed, primarily in the afternoon, highlighting the need to adjust kickoff times (9). The third was the assessment of event feasibility in the context of future climate change. A study of school sports clubs in Japan reported that the time windows available for outdoor sports activities will decrease further in the future and that introducing early morning practices or reducing outdoor training alone will not be sufficient measures (10).

Many previous studies have evaluated heat risk using environmental indices such as WBGT. However, environmental indices alone do not directly indicate whether a tournament schedule can be completed, how many matches can be conducted, or how much operational margin remains under hot conditions. Tournament organizers make their final decisions based not on environmental indicators themselves, but on the feasibility of holding the event under heat stress conditions. Therefore, a quantitative framework is needed to translate environmental heat conditions into operational indicators relevant to tournament management.

School sports club tournaments in Japan are often held nationwide during the summer vacation period, due to the scheduling of the academic curriculum, and matches are concentrated during the period of the year when heat stress is particularly high. Furthermore, depending on the sport, tournaments are often held outdoors for long periods, with multiple matches taking place in a single day, making them susceptible to the effects of hot environments. Tennis is a sport in which hot environments can directly affect tournament operations, as match durations vary, and multiple matches are held outdoors in a single day. Top-level competitions, such as nationwide school sports tournaments and the All-Japan Junior Championships, are held in August—the height of summer. Consequently, athletes must advance through qualifying rounds while contending with the added physical strain of rising temperatures, and then compete in the national tournaments amid grueling schedules in the sweltering heat.

According to the Japan Sports Association guidelines for heatstroke prevention, a WBGT of 28 or higher is classified as a “severe warning” level and is designated as the threshold at which strenuous exercise should be suspended (11). The Japan Tennis Association heat-safety guidelines stipulate that measures for cooling the body surface must be provided when the WBGT exceeds 28 (12). The risk of heatstroke is approximately six times higher at WBGT levels of 28–29 than at 25–26 (13), and the number of fatalities increases when the WBGT exceeds 28 (14). However, heat-related thresholds vary among organizations and sports, and recent evidence has highlighted the importance of considering sport-specific and environmental conditions when establishing such thresholds, including more conservative criteria for sports associated with higher heat-illness risk, such as tennis (15). In the present study, WBGT <28 was therefore not regarded as a universal criterion for competition suspension but was operationally defined as a lower-risk condition for standardized pre-tournament evaluation based on the above guidelines and epidemiological evidence. The cumulative time during which WBGT remained below 28 was defined as “low-risk operational time.” The objective of this study was to develop and apply a framework that translates WBGT-based environmental conditions into operational summer national (top-level) tournament-management indicators, including low-risk operational time, feasible tournament days, match capacity, and operational margin, thereby providing decision-makers with information directly relevant to tournament planning and management.

## 2 Methods

### 2.1 Study design

As tournament organizers must determine whether scheduled matches can be completed under heat-related restrictions, we translated WBGT-based environmental conditions into operational indicators relevant to tournament management. Using nationwide WBGT observations, we evaluated tournament feasibility with respect to available playing time and match capacity across different scheduling scenarios.

### 2.2 WBGT data and sunset time collection

WBGT data were obtained from the Ministry of the Environment (16). At the time of data collection, black globe temperature, a component of WBGT, was directly measured at only 11 Japan Meteorological Agency observation stations nationwide. Therefore, to ensure a consistent assessment based on directly measured black globe temperature, these 11 stations were selected: Sapporo [43°N], Sendai [38°N], Tokyo [37°N], Niigata [35°N], Nagoya [35°N], Osaka [34°N], Hiroshima [34°N], Kochi [33°N], Fukuoka [33°N], Kagoshima [31°N], and Naha [26°N]. WBGT data were collected from April 1 to October 31 for the years 2018–2022. The natural wet-bulb temperature (Tnw), a component of WBGT, is not measured directly at the Japan Meteorological Agency observation stations; instead, it is calculated and estimated from the dry-bulb temperature, dew point temperature, and atmospheric pressure using the method of Iribarne and Godson (17). Additionally, using the “Koyomi station (18)” provided by the National Astronomical Observatory of Japan, sunset times for the 11 WBGT observation station locations nationwide were also collected for the same period. As the WBGT service provides data on an hourly basis, the latest available WBGT data before the average sunset time were used as the WBGT at sunset. Furthermore, because this study conducted analyses assuming changes in tournament timing, in addition to the standard period from 09:00 to sunset, WBGT data from the early morning (07:00) and evening (20:00) were also included in the analysis.

### 2.3 Low-risk operational time definition

In this study, based on the Japan Sports Association heatstroke prevention guidelines (11), the cumulative duration with WBGT <28 was defined as low-risk operational time. For each day, the number of hours with WBGT <28 was summed to obtain the daily low-risk operational time. To enable a comparison across conditions, the low-risk operational time was divided by the total number of observation hours per day to calculate the day-equivalent feasible days. Low-risk operational time was converted into the number of matches that could be completed because tournament success is ultimately determined by whether scheduled matches can be finished. This approach allowed for environmental heat conditions to be interpreted in terms directly relevant to tournament operation.

### 2.4 Statistical analysis

#### 2.4.1 Descriptive analysis

The number of feasible tournament days was calculated for each location by year and month, and the feasibility distribution was evaluated.

#### 2.4.2 Regression analysis

A binomial logistic regression model was used to evaluate the association of tournament dates and timing conditions with the occurrence of WBGT <28. Each hourly observation was treated as a binary outcome (WBGT <28 vs. ≥ 28). The number of hours with WBGT <28 was modeled as the outcome, with the total number of observed hours under each condition specified as the binomial denominator. Tournament conditions (dates and timing) were included as explanatory variables and observation locations were included as adjustment variables. The analysis was restricted to July–September, when heat stress was the highest. The tournament dates were categorized into early (1st–10th), middle (11th–20th), and late (21st–end) periods. The timing conditions included a standard model (09:00 to sunset). In addition, early morning models (07:00–12:00 and 15:00 to sunset) and nighttime models (09:00–12:00 and 15:00–20:00) were established to avoid periods of peak heat exposure. Model estimates were expressed as odds ratios (ORs) with 95% confidence intervals (CIs). The ORs represent the odds of WBGT being below 28 according to tournament dates and timing conditions. Statistical analyses were performed using SPSS version 28.0 (IBM Japan, Tokyo, Japan), with significance set at *p* < 0.05.

#### 2.4.3 Scenario analysis

Multiple scenarios were built using regression analysis to assess tournament feasibility. The baseline was “early August × standard time slot (09:00 to sunset),” mirroring typical school tennis schedules. Building on the scenario construction described above, the low-risk operational time for each scenario was estimated by applying the relative effects of tournament dates and timing derived from the binomial logistic regression model to the baseline condition. Specifically, the baseline proportion of low-risk operational time was calculated by dividing the baseline low-risk operational time by the baseline daily observation time. The estimated ORs were then applied to the corresponding baseline odds, and the resulting values were converted back to proportions to estimate low-risk operational time under each scenario.

The improvement effect was expressed as *Δ*Time, defined as the difference in estimated low-risk operational time relative to the baseline condition. *Δ*Time was then divided by the baseline daily observation time to compute the corresponding change in day-equivalent feasible days (*Δ*Days). For interpretability, *Δ*Days was also expressed relative to a 7-day tournament period. For scenarios combining tournament dates and timing, the variance of the combined estimates was computed using the variance–covariance matrix of the regression coefficients. The corresponding 95% CIs were then transformed into *Δ*Days using the same procedure as that used for the point estimates.

#### 2.4.4 Feasibility threshold definition

To assess tournament feasibility, we used the first day of the individual tennis tournament at the 2025 Inter-High School Athletic Meet as a model case and defined the feasibility thresholds based on the low-risk operational time required to complete the scheduled matches. Thresholds were established under the assumptions of a one-set match duration of 30–40 min and the use of 20 courts.

According to the official schedule (19), 180 matches are played per day. Under the theoretical minimum conditions (30 min per match with no intervals), the total time required was 90 h (180 matches   ×   30 min), corresponding to 4.5 h per court. This value was defined as the theoretical minimum threshold. Under more realistic conditions, assuming a 30-min match duration with a 10-min interval between matches (40 min per match), the required low-risk operational time was 6.0 h, which was defined as the operational feasibility threshold. Furthermore, assuming a 40-min match duration with a 10-min interval (50 min per match), the required low-risk operational time was increased to 7.5 h, which was defined as the extended feasibility threshold. These thresholds were introduced to evaluate whether the required number of matches could be completed within the available low-risk operational time. Using these three thresholds, the tournament feasibility under each scenario was evaluated based on whether the estimated low-risk operational time met or exceeded each threshold. To further quantify the magnitude of the surplus or deficit relative to the operational feasibility threshold, the operational margin and match surplus were calculated. Operational margin was defined as the difference between the mean daily low-risk operational time and the operational feasibility threshold (6.0 h/day). Positive values indicate a surplus relative to the operational feasibility threshold, whereas negative values indicate a deficit. Match surplus was calculated as the difference between the estimated match capacity and the required capacity of nine matches per court per day, based on a 30-min match duration and a 10-min interval between matches. These indicators were used to quantify the operational margin available under each tournament scenario.

## 3 Results

### 3.1 Distribution of feasible tournament days according to season and observation site

In this study, 119,048 WBGT observations were analyzed after excluding 20 records with missing data. Figure 1 shows the distribution of feasible tournament days by location and month. Feasible days decreased from July through September across all locations, with the lowest values generally observed in August.

**Figure 1:**
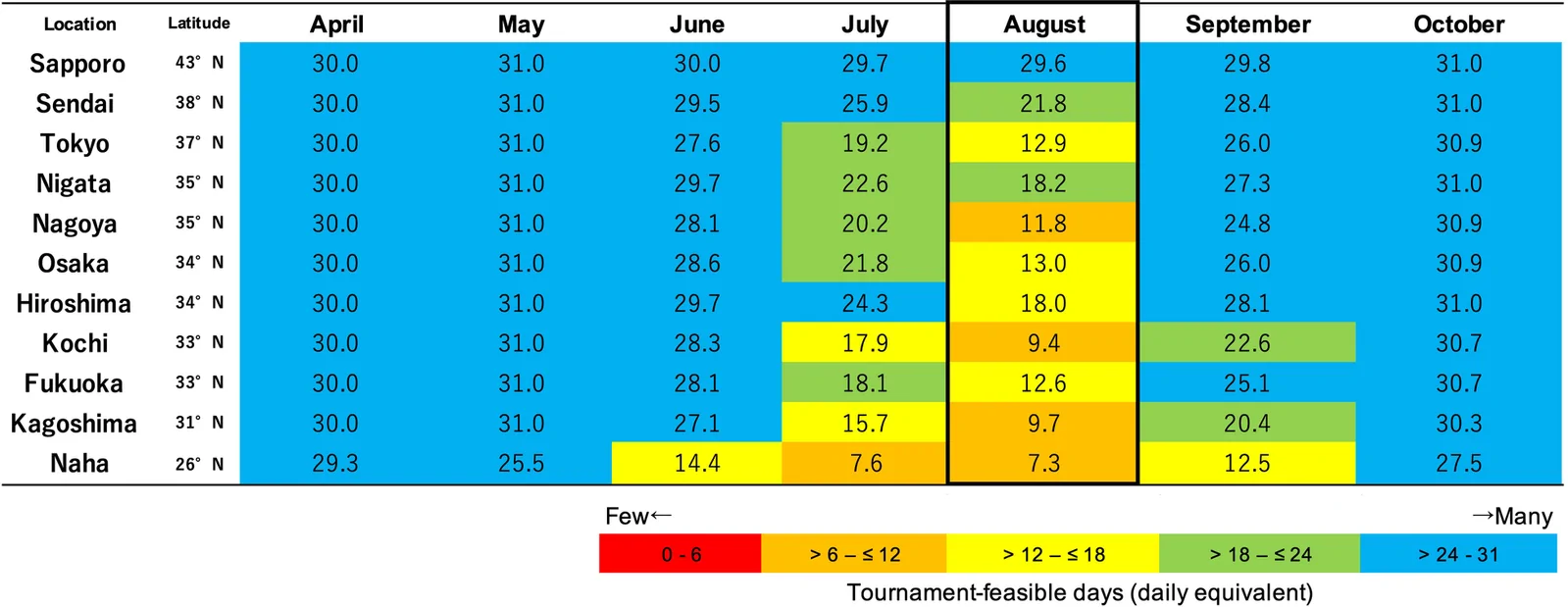
Distribution of tournament-feasible days across months and locations. Values represent aggregated data from 2018 to 2022 and are expressed as day-equivalent values derived from cumulative WBGT <28 h between 09:00 and sunset.

### 3.2 Impact of tournament conditions (dates and timing)

Figure 2 shows the associations of tournament dates and timing conditions with the odds of WBGT being below 28. Compared with early August (reference period), the odds of WBGT being below 28 were higher in mid-July (OR = 1.19, 95% CI: 1.14–1.25) and late July (OR = 1.11, 95% CI: 1.06–1.16). In contrast, the odds were lower throughout September, with the lowest OR observed in early September (OR = 0.75, 95% CI: 0.72–0.79). Regarding timing, compared with the standard model (09:00 to sunset), the odds of WBGT being below 28 were higher in both the nighttime model (OR = 1.46, 95% CI: 1.42–1.50) and the early morning model (OR = 1.36, 95% CI: 1.33–1.40). Mean sunset times were earlier in September than in August at all 11 locations, with differences ranging from 31 min in Naha to 51 min in Sapporo.

**Figure 2:**
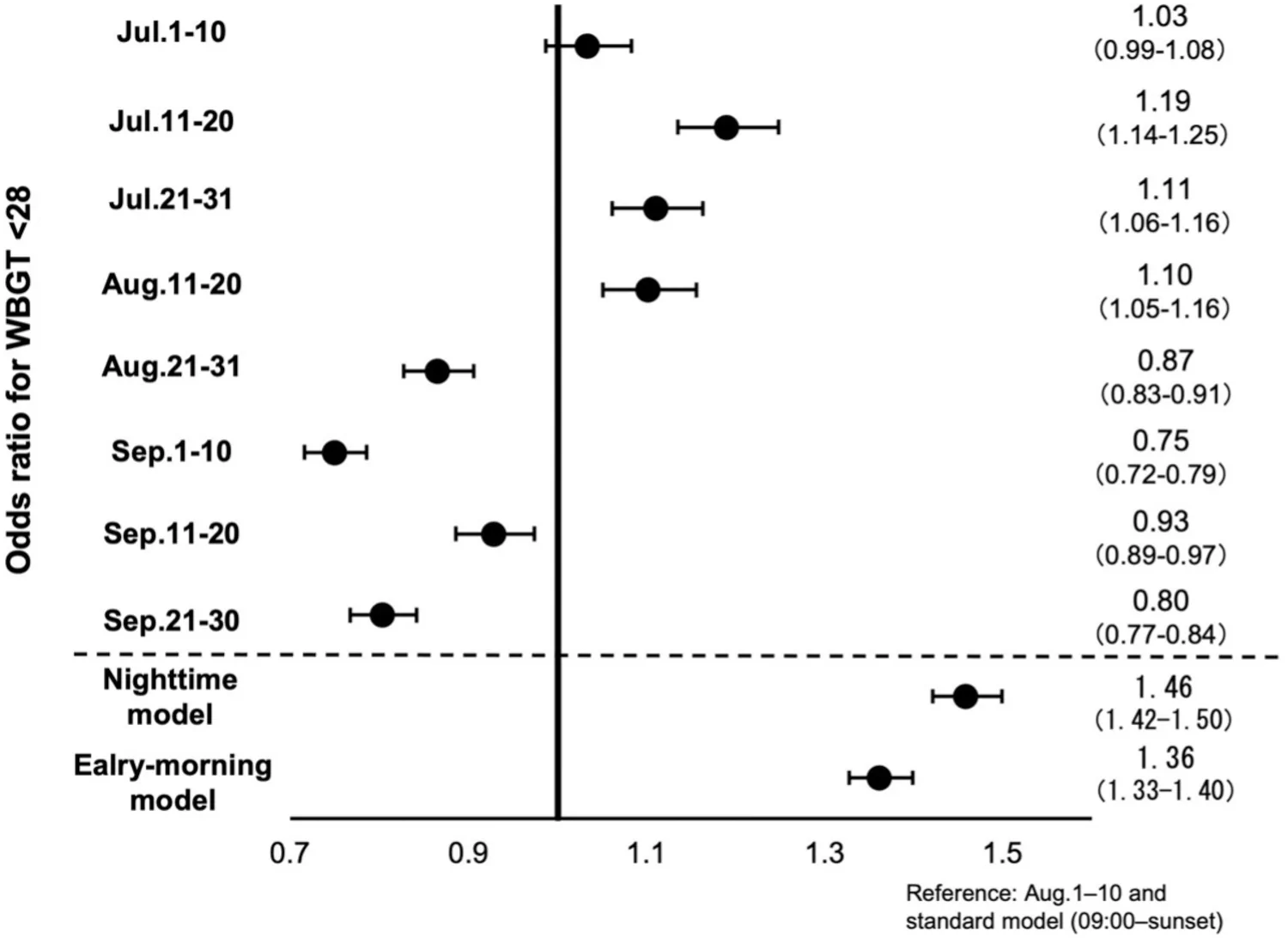
Odds ratios (ors) and 95% confidence intervals (cis) for WBGT being below 28 by tournament dates and timing conditions. ORs and 95% CIs were estimated using a binomial logistic regression model. Early August and the standard model (09:00 to sunset) were used as the reference categories.

### 3.3 Impact of different combinations of tournament conditions

Figure 3 shows the results of the scenario analysis combining different dates and timing conditions. Under the baseline conditions (early August × standard model), the average daily observation time was 10.3 h, of which 6.7 h was defined as low-risk operational time (WBGT <28). The increase in low-risk operational time was greater when timing adjustments were combined with changes in tournament dates than when tournament dates alone were changed. When expressed as the equivalent change over a 7-day tournament period, the greatest increase in low-risk operational time was approximately 0.8 days under the mid-July × nighttime model.

**Figure 3:**
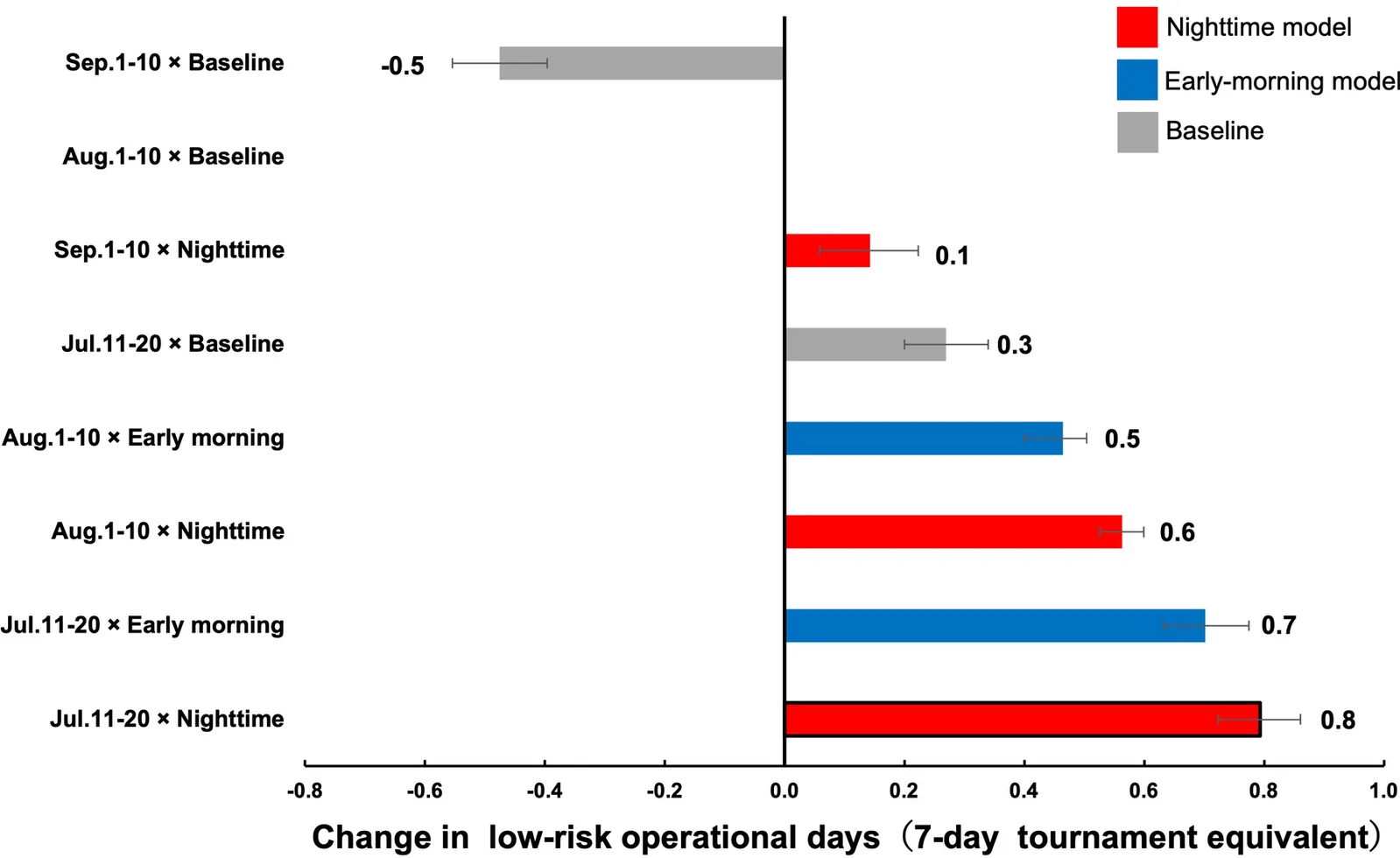
Changes in low-risk operational days under combined scheduling scenarios. Values represent differences in day-equivalent values relative to the baseline condition (August 1–10, 09:00 to sunset), expressed as a 7-day tournament equivalent. Error bars indicate 95% CIs. The August 1–10 baseline scenario was the reference condition (*Δ* days = 0).

### 3.4 Evaluation based on feasibility thresholds

Figure 4 shows the proportion of scenarios in which the low-risk operational time met the operational feasibility threshold (6.0 h). Even under the same dates, the timing adjustments increased the proportion of scenarios that met the threshold.

**Figure 4:**
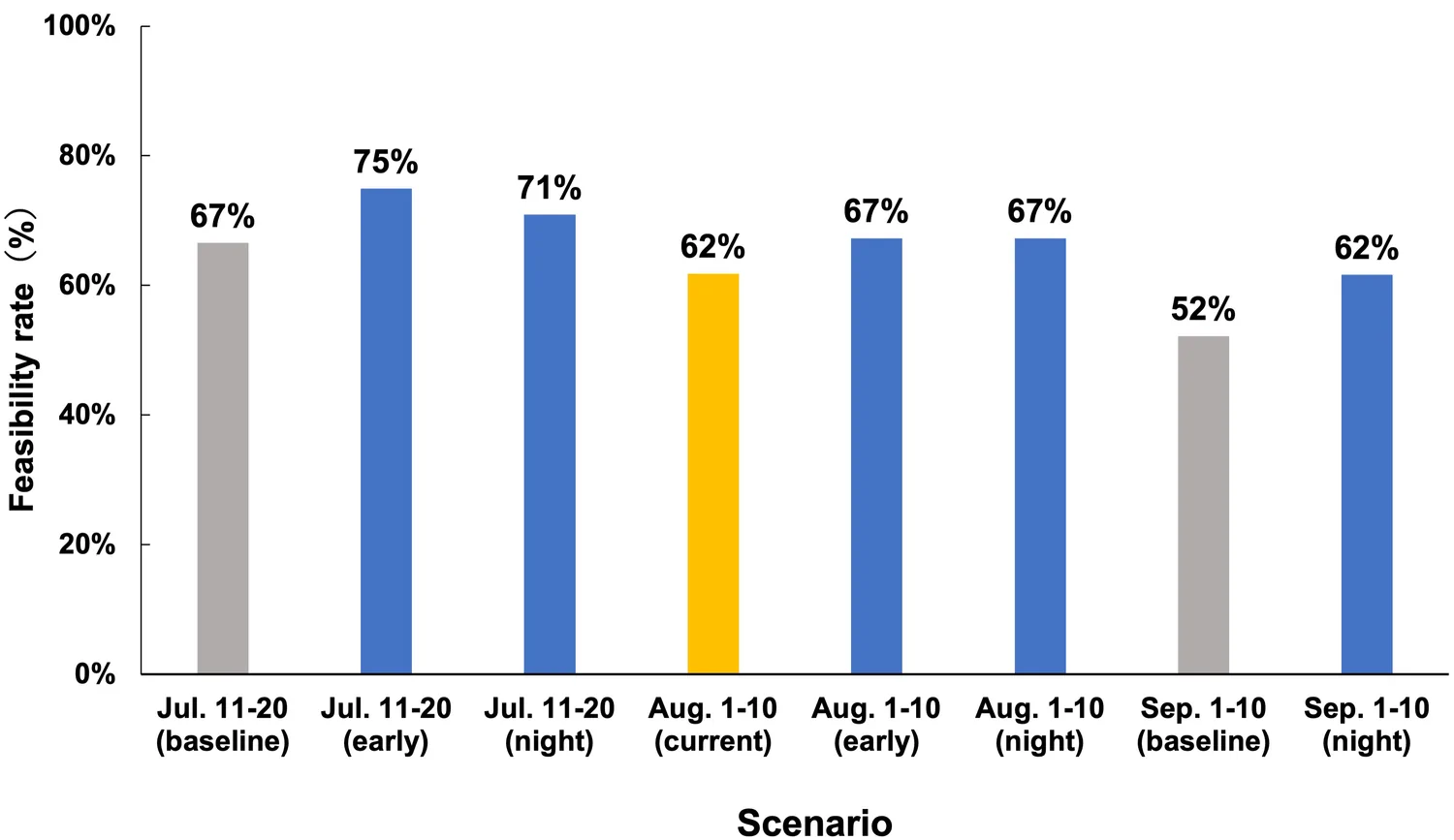
Proportion of days meeting the operational feasibility threshold under each scenario. Values indicate the percentage of days with ≥6.0 h of WBGT < 28 under each condition.

Figure 5 presents the operational margin and match surplus relative to the operational feasibility threshold. The largest mean operational margin was observed in the July 11–20 early-morning scenario (+2.2 h/day), followed by the July 11–20 nighttime scenario (+1.6 h/day). The September 1–10 baseline scenario showed a negative mean margin (−0.5 h/day), indicating that mean low-risk operational time did not meet the operational feasibility threshold. A similar pattern was observed for match surplus. The July 11–20 early-morning scenario showed the largest mean match surplus (+3.3 matches per court per day), whereas the September 1–10 baseline scenario showed a mean deficit (−0.8 matches per court per day). Although many scenarios met the operational feasibility threshold, the amount of operational margin varied substantially among scenarios. These findings indicate that threshold attainment alone does not fully characterize tournament feasibility, and that operational margin provides additional information on the degree of surplus or deficit relative to the operational feasibility threshold.

**Figure 5:**
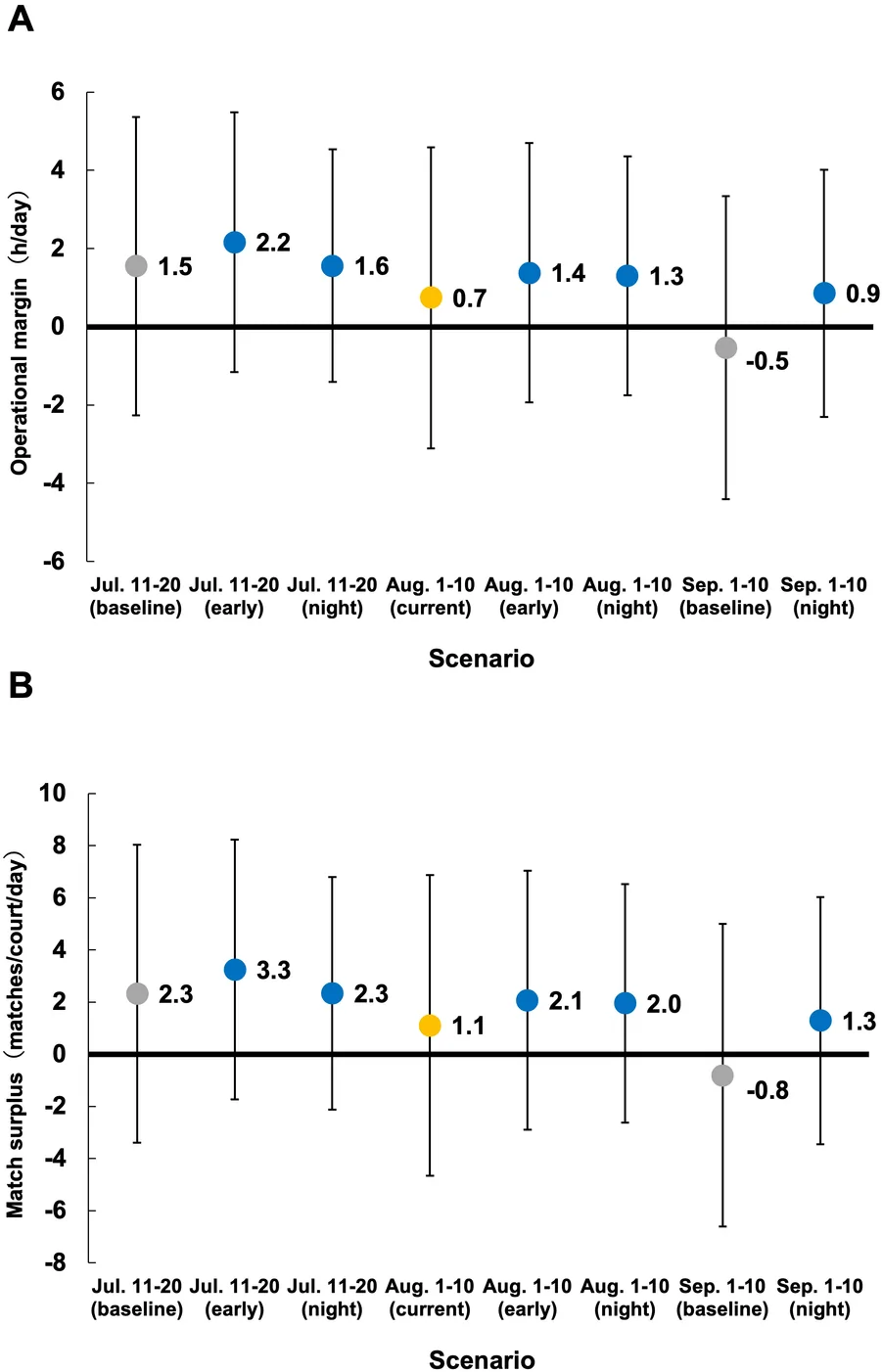
Operational margin and match surplus across tournament scheduling scenarios. **(A)** Operational margin was calculated as the difference between the mean daily low-risk operational time and the predefined operational feasibility threshold of 6.0 h/day. **(B)** Match surplus was calculated as the difference between estimated match capacity and the required capacity of 9 matches per court per day. Points represent mean values, and error bars indicate SDs derived from the distribution of daily low-risk operational time within each scenario. Positive values indicate a surplus relative to the operational feasibility threshold, whereas negative values indicate a deficit. The horizontal line at zero indicates the point at which available low-risk operational time equals the predefined operational feasibility threshold.

## 4 Discussion

In this study, we evaluated tournament feasibility in hot environments using WBGT-based low-risk operational time as an indicator. The results showed substantial seasonal variation in tournament feasibility, while timing adjustments consistently improved feasibility, and the effects of shifting tournament dates were more limited. The greatest improvement was observed when both the factors were combined. However, although tournaments were feasible under many conditions, their operational margins remained limited. Rather than evaluating WBGT values alone, we translated environmental conditions into operational outcomes, including low-risk operational time and match capacity, because tournament organizers make decisions based on scheduling feasibility rather than environmental indices themselves.

Shifting tournament dates had a smaller effect on tournament feasibility than timing adjustments. Although moving the tournament to July improved feasibility, postponing it to September reduced it. This reduction may partly be attributable to the earlier sunset in September. Across the 11 locations, mean sunset times in September were approximately 31–51 min earlier than those in August. Because WBGT data were available at hourly intervals and we used the latest available WBGT value before sunset, earlier sunsets could shorten the observation period under sunset-defined timing conditions. This applied to both the standard model (09:00 to sunset) and the afternoon component of the early-morning model (07:00–12:00 and 15:00 to sunset), whereas the nighttime model (09:00–12:00 and 15:00–20:00) was not constrained by sunset time. Thus, the effect of shifting tournament dates on feasibility may reflect not only seasonal variation in WBGT but also differences in how seasonal changes in sunset time affect the observation period under different tournament timing conditions. These findings suggest that adjustments to tournament timing may influence tournament feasibility more than date changes defined by 10-day intervals.

Timing adjustments were the most consistent factor for improving feasibility. Heat stress and performance decrements in outdoor environments are strongly influenced by solar radiation (20) and shifting activity to earlier times of day has been shown to reduce heat stress in endurance sports (8). In the present study, periods with WBGT <28 were concentrated in the morning and evening, and low-risk operational time increased when these periods were utilized while avoiding midday. These findings are consistent with previous studies and suggest that adjusting the time of day may reduce reliance on periods of higher heat exposure while increasing low-risk operational time.

The greatest improvement in tournament feasibility was observed when multiple conditions, such as dates and timing, were combined, compared with changes to a single factor. These findings indicate that adjusting a combination of conditions is more effective than relying on a single measure. While existing heat policies developed by international federations have primarily focused on determining whether tournaments should proceed and on-site responses, that is, risk classification and management (21), this study extends this perspective by incorporating heat environment evaluation into tournament planning and introducing “operational margin” as a practical framework for decision-making. Operational margin represents the difference between the available low-risk operational time and the operational feasibility threshold, thereby quantifying the degree of temporal surplus or deficit under each tournament condition.

Many scenarios met the operational feasibility threshold; however, the magnitude of the remaining operational margin varied across tournament conditions. Moreover, the relatively large SDs indicate substantial variability in daily operational margin within each scenario. The mean operational margin provides a standardized basis for comparing alternative tournament conditions, whereas the observed variability indicates that these average estimates should be interpreted cautiously when applied to specific tournament settings. The operational margin and match surplus required in actual tournaments remain difficult to define because match duration and operational delays can vary across matches and tournaments. Therefore, the operational margin and match surplus values in this study should not be interpreted as prescriptive thresholds indicating that a specific margin is sufficient to complete a tournament. Nevertheless, these indicators provide information beyond a binary assessment of whether the operational feasibility threshold is met by quantifying the magnitude of the surplus or deficit relative to the threshold. For example, under the early-August condition, the standard model provided an operational margin of +0.7 h/day (match surplus: +1.1 matches/court/day), whereas the early-morning model increased these values to +1.4 h/day and +2.1 matches/court/day, respectively. Thus, even under conditions in which the operational feasibility threshold was met on average, the magnitude of the operational margin differed between tournament designs. These findings suggest that the magnitude of the operational margin and match surplus should also be considered when comparing alternative tournament designs.

Furthermore, existing heat-safety guidelines (12) primarily emphasize environmental monitoring and on-site responses. The present findings suggest that operational indicators such as operational margin and match surplus may complement environmental metrics by indicating the degree of temporal margin available during tournament planning. Future studies should collect data from actual tournaments to determine the operational margin and match surplus needed to accommodate variability in match duration and tournament operations. Such evidence could eventually support the development of recommended reference values for operational margin and match surplus in heat-management guidelines for tournament organizers, providing practical benchmarks for pre-tournament planning and decision-making.

These findings suggest that advancing the tournament dates and adopting a split-day format that avoids peak afternoon hours may increase low-risk operational time and improve tournament feasibility. However, several challenges must be addressed before implementing these strategies in school sports. Adjusting the tournament dates requires coordination with the school curricula and other championships. In Japan, regional differences in summer vacation length have been reported due to climatic variations (22), and substantially advancing dates may lead to conflicts with academic activities. In addition, early morning and nighttime schedules may increase operational costs (e.g., lighting) and pose challenges for student safety management. Nevertheless, a project team has recently been established within the Inter-High framework to review event formats (23), and the findings of this study may provide a useful evidence base for these discussions.

The results of this study indicate that the current tournament schedule (09:00 to sunset in early August) may provide only a limited operational margin under the standardized tournament conditions. This finding suggests that, under severe heat conditions, modifications to tournament dates or timing alone may not always provide sufficient flexibility in tournament scheduling. To prioritize student safety above all else, it may be necessary to go beyond mere adjustments to the tournament dates or timing and consider alternative match formats—such as the time-limit system used in the Ultimate Tennis Showdown (UTS) (24), which divides matches by time rather than number of games, or the short-set format—exclusively for summer tournaments. Such formats may improve the predictability of match durations and may facilitate tournament operations within the limited range of “low-risk operational time” (WBGT <28). Further research is needed to determine whether such alternative measures can enhance schedule predictability in severe heat environments and increase the operational margin available under low-risk conditions.

As demonstrated by the nationwide analysis, tournament feasibility varied substantially according to seasonal conditions. These findings suggest that maintaining a uniform nationwide tournament schedule under increasingly hot summer conditions may become progressively more challenging. Although the present study did not evaluate alternative tournament calendars, the results highlight the need for future discussions regarding climate-adaptive scheduling strategies, including potential modifications to tournament timing and seasonal scheduling frameworks. At the same time, changes to the timing of national tournaments should not be considered independently of regional qualifying tournaments. Delaying or advancing a national tournament may require corresponding changes to regional tournament dates, potentially shifting heat exposure to these events. Therefore, schedule modifications should be considered within the broader tournament calendar, including regional qualifying competitions, rather than focusing solely on the national tournament. Such discussions may become increasingly important if future climate conditions further limit the availability of lower-risk operational periods for outdoor sports during summer. Researchers have also examined adaptation strategies involving changes to when and where competitions are held in other major international sporting events. Mullan et al. (2025) highlighted the potential importance of rescheduling kick-off times outside peak afternoon heat at the 2026 FIFA World Cup (9), whereas Oyama et al. (2022) showed that changing the season and host location could improve the feasibility of hosting the Olympic marathon under future climate conditions (25). Together with the present findings, these studies suggest that heat adaptation in sporting events may require consideration not only of on-site heat management but also of when and where competitions are held. Other potential measures in tennis include installing court lighting to extend playable hours and holding tournaments in cooler regions. Facilities that reduce direct solar exposure, such as indoor courts, may also warrant consideration.

This study had three limitations. First, it was based on WBGT data from Japan Meteorological Agency stations (11 locations) and may not have fully captured the microclimatic and surface effects of actual tournament environments. These stations are installed over grass surfaces to reduce ground heat, whereas tournament settings may be influenced by factors such as ground radiation, leading to different WBGT (26, 27). Future studies should incorporate on-site measurements to better characterize thermal conditions in real competitive environments. Second, the observation location was treated as an adjustment variable, and the analysis focused on nationwide average trends; therefore, region-specific climatic characteristics were not examined. Third, the present evaluation was based on the predefined criterion of WBGT <28 for low-risk operational conditions. Heat-related thresholds for activity modification or suspension vary among governing organizations, participant populations, and sports, and sport-specific adjustment of heat-safety thresholds has also been proposed (15). Therefore, the present findings should be interpreted as tournament feasibility under the predefined WBGT <28 condition rather than as evidence that this threshold is universally applicable to tournament management. Future studies should examine how tournament feasibility, operational margin, and match surplus may differ when alternative or sport-specific thresholds are applied. Despite these limitations, this study is notable in that it quantifies the low-risk operational time based on the WBGT and provides a comprehensive evaluation of the influence of dates and timing on tournament feasibility. Furthermore, by translating WBGT-based low-risk operational time into practical indicators such as operational margin and match surplus, this study provides additional information on the degree of surplus or deficit under different tournament conditions. More importantly, this study demonstrated that translating WBGT-based environmental conditions into operational indicators can reveal operational vulnerabilities that are not apparent from environmental indices alone, thereby bridging the gap between environmental monitoring and practical tournament management.

## 5 Conclusions

To the best of our knowledge, this is the first study to move beyond conventional heat-risk assessment and translate WBGT-based environmental conditions into operational indicators directly relevant to tournament management. The results showed that timing adjustments substantially increased low-risk operational time (WBGT <28), whereas shifting the tournament dates alone had limited effects. Furthermore, combining date and timing adjustments produced the greatest improvements in feasibility. Although many conditions met the operational feasibility threshold on average, the operational margin varied across tournament conditions. These findings suggest that tournament feasibility should be evaluated not only by whether the operational feasibility threshold is met but also by the magnitude of the operational margin available. More importantly, this study demonstrates a framework for bridging environmental heat monitoring and tournament management by translating heat exposure into operational outcomes. This approach provides decision-makers with information directly relevant to tournament planning, rather than relying solely on environmental heat indices. This framework may also provide a foundation for future discussions on climate-adaptive tournament scheduling and management.

## Data Availability

Publicly available datasets were analyzed in this study. This data can be found here: https://www.wbgt.env.go.jp/wbgt_data.php.
